# Interleukin-13 Inhibits Lipopolysaccharide-Induced BPIFA1 Expression in Nasal Epithelial Cells

**DOI:** 10.1371/journal.pone.0143484

**Published:** 2015-12-08

**Authors:** Yung-An Tsou, Chia-Der Lin, Hui-Chen Chen, Hui-Ying Hsu, Lii-Tzu Wu, Chuan Chiang-Ni, Chih-Jung Chen, Tsu-Fang Wu, Min-Chuan Kao, Yu-An Chen, Ming-Te Peng, Ming-Hsui Tsai, Chuan-Mu Chen, Chih-Ho Lai

**Affiliations:** 1 Department of Otolaryngology-Head and Neck Surgery, China Medical University and Hospital, Taichung, Taiwan; 2 Department of Life Sciences, National Chung Hsing University, Taichung, Taiwan; 3 Graduate Institute of Basic Medical Science, School of Medicine, China Medical University and Hospital, Taichung, Taiwan; 4 Department of Microbiology and Immunology, Graduate Institute of Biomedical Sciences, Chang Gung University, Taoyuan, Taiwan; 5 Division of Paediatric Infectious Diseases, Department of Paediatrics, Chang Gung Children's Hospital and Chang Gung Memorial Hospital, Taoyuan, Taiwan; 6 Department of Applied Cosmetology, Hung Kuang University, Taichung, Taiwan; 7 Department of Nursing, Asia University, Taichung, Taiwan; University of Pittsburgh, UNITED STATES

## Abstract

Short palate, lung, and nasal epithelium clone 1 (SPLUNC1) protein is expressed in human nasopharyngeal and respiratory epithelium and has demonstrated antimicrobial activity. SPLUNC1 is now referred to as bactericidal/permeability-increasing fold containing family A, member 1 (BPIFA1). Reduced BPIFA1 expression is associated with bacterial colonization in patients with chronic rhinosinusitis with nasal polyps (CRSwNP). Interleukin 13 (IL-13), predominately secreted by T helper 2 (T_H_2) cells, has been found to contribute to airway allergies and suppress BPIFA1 expression in nasal epithelial cells. However, the molecular mechanism of IL-13 perturbation of bacterial infection and BPIFA1 expression in host airways remains unclear. In this study, we found that lipopolysaccharide (LPS)-induced BPIFA1 expression in nasal epithelial cells was mediated through the JNK/c-Jun signaling pathway and AP-1 activation. We further demonstrated that IL-13 downregulated the LPS-induced activation of phosphorylated JNK and c-Jun, followed by attenuation of BPIFA1 expression. Moreover, the immunohistochemical analysis showed that IL-13 prominently suppressed BPIFA1 expression in eosinophilic CRSwNP patients with bacterial infection. Taken together, these results suggest that IL-13 plays a critical role in attenuation of bacteria-induced BPIFA1 expression that may result in eosinophilic CRSwNP.

## Introduction

Short palate, lung, and nasal epithelium clone 1 (SPLUNC1) protein, a member of the bactericidal/permeability-increasing protein (BPI) family, is expressed in human nasopharyngeal and respiratory epithelium [1,2] and is also referred to as BPI fold containing family A, member 1 (BPIFA1) [3]. Several studies have shown that BPIFA1 possesses antimicrobial activity [4,5]. Additionally, BPIFA1 exhibits surfactant properties of airway secretions [6], and this activity may inhibit biofilm formation of the bacteria [7]. It has also been reported that BPIFA1 plays an important role in the regulation of airway surface liquid volume [8]. Reduced BPIFA1 expression may contribute to the persistent nature of bacterial infections in airways, suggesting that BPIFA1 may serve as a host defense protein against bacterial infection [5,9]. In a recent report, we analyzed patients who underwent sinus surgery for chronic rhinosinusitis with nasal polyps (CRSwNP) and found that reduced BPIFA1 expression was associated with bacterial colonization and negative treatment outcomes in these patients [10]. This evidence indicated that decreased BPIFA1 expression might facilitate bacterial infection in a host, leading to severe disease manifestations.

Patients with CRSwNP generally require revision sinus surgery for persistent nasal disease [11,12]. CRSwNP is a disorder characterized by the development of T_H_2 inflammation and tissue eosinophilia that may be induced by microbial infections [13]. Interleukin 13 (IL-13), a cytokine predominately secreted by T_H_2, has been found to contribute to airway allergies and to suppress BPIFA1 expression in nasal epithelial cells [14]. Additionally, lipopolysaccharide (LPS), which is secreted from bacterial cell walls and serves as a Toll-like receptor 4 (TLR-4) agonist, has been found to upregulate BPIFA1 expression in polyp epithelial cells from patients with eosinophilic CRSwNP [15]. These findings indicate that IL-13 plays a critical role in regulation of BPIFA1 expression in patients with eosinophilic CRSwNP. However, the molecular mechanisms underlying IL-13 perturbation of bacterial infection and BPIFA1 expression in host airways require further exploration.

Considering the potential role of BPIFA1 in host innate immunity, we established an *in vitro* human nasal cell model and examined patient tissues to determine whether LPS could upregulate BPIFA1 expression. We then demonstrated that IL-13 downregulated LPS-induced activation of phosphorylated JNK and c-Jun, followed by attenuation of BPIFA1 expression. Our results provide insight into the molecular mechanisms underlying the function of BPIFA1, which is modulated by the immune response and can be counteracted in a persistent infection in host airways.

## Materials and Methods

### Antibodies and reagents

Antibodies against β-actin, BPIFA1 (SPLUNC1), and phospho-JNK were purchased from Santa Cruz Biotechnology (Santa Cruz, CA, USA). Monoclonal antibody specific to BPIFA1 (MAB1897) was purchased from R&D Systems (Minneapolis, MN, USA). Antibodies specific for phospho-c-Jun (Ser63) and phospho-p38 MAPK (Thr180/Tyr182) were purchased from Cell Signaling (Danvers, MA, USA). Anti-phospho-Erk1/2 (Thr180/Tyr182) antibody was purchased from Millipore (Billerica, MA, USA). SB203580 (p38 inhibitor), PD98059 (ERK inhibitor), and SP600125 (JNK inhibitor) were purchased from Calbiochem (San Diego, CA, USA). 4',6-Diamidino-2-phenylindole (DAPI) was purchased from Molecular Probes (Invitrogen, Carlsbad, CA, USA). Human recombinant interleukin 13 (IL-13) was purchased from Sigma-Aldrich (St. Louis, MO, USA). JNK-dominant negative mutant and AP-1 luciferase reporter were kindly provided by Dr. Chih-Hsin Tang (China Medical University) [16]. All other reagents and chemicals were purchased from Sigma-Aldrich.

### Cell culture and treatment

Human nasal septum squamous carcinoma RPMI-2650 cells (ATCC CCL-30) were cultured in modified Eagle’s medium (MEM; Gibco, Grand Island, NY, USA) supplemented with 10% fetal bovine serum (FBS, HyClone, Logan, UT, USA), 1% penicillin-streptomycin, 1% non-essential amino acid, and 1% sodium pyruvate, at 37°C in an atmosphere containing 5% CO_2_. Cells were treated with various concentrations (0–20 μg/ml) of LPS for 2 h, and mRNA levels of BPIFA1 were analyzed by quantitative real-time PCR. Cell lysates were prepared to measure BPIFA1 protein expression levels by western blot after incubation with LPS (0–20 μg/ml) for 24 h. Protein expression levels of BPIFA1 were quantified by densitometric analysis. For protein expression analysis, the cells were pretreated with inhibitors (PD98059, SP600125, or SB203580 at the concentrations of 20 μM, 10 μM, and 20 μM, respectively) for 1 h and then treated with 10 μg/ml lipopolysaccharide (LPS; *Escherichia coli* 055:B5, Sigma-Aldrich) for additional 2 h.

### Western blot analysis

Whole cell lysates were prepared as described previously [17]. Proteins were separated by sodium dodecyl sulfate polyacrylamide gel electrophoresis (SDS-PAGE) and transferred to polyvinylidene difluoride membranes (Millipore, Billerica, MA, USA). Membranes were incubated with 5% skim milk at room temperature for 1 h, probed with primary antibodies as indicated, and incubated with horseradish peroxidase–conjugated secondary antibodies (Millipore). The proteins of interest were detected and visualized using enhanced chemiluminescence and Kodak X-OMAT LS film (Eastman Kodak, Rochester, NY, USA) as described previously [18].

### Reverse transcription and real-time quantitative PCR

Total RNA from RPMI-2650 cells was isolated using TRIzol (Invitrogen), as described previously [19]. Briefly, total RNA (1 μg) was reverse-transcribed into cDNA by using oligo(dT) primers and Moloney Murine Leukemia Virus reverse transcriptase (Invitrogen). The RNA expression levels of BPIFA1 were determined by reverse transcription quantitative PCR (RT-qPCR), using StepOnePlus^™^ Real-Time PCR Systems (Applied Biosystems, Foster City, CA, USA). The oligonucleotide primers used corresponded to human BPIFA1 (forward, 5′-CTTGGCCTTGTGCAGAGC-3′; and reverse, 5′-CAACAGACTTGCACCGACC-3′) and glyceraldehyde-3-phosphate dehydrogenase (GAPDH; forward, 5′-CCCCCAATGTATCCGTTGTG-3′; and reverse, 5′-TAGCCCAGGATGCCCTTTAGT-3′). All oligonucleotide primers were synthesized by Invitrogen. After pre-incubation at 50°C for 2 min and 95°C for 10 min, PCR was performed with 40 cycles of 95°C for 15 s and 60°C for 60 s. The threshold was set above the non-template control background and within the linear phase of target gene amplification in order to calculate the cycle number at which the transcript was detected (denoted as [*C*_*T*_]).

### Reporter activity assay

RPMI-2650 cells were grown to 90% confluence and transfected with AP-1-Luc reporter by using Lipofectamine 2000 (Invitrogen). After a 24-h incubation, the transfected cells were treated with curcumin (10 μM) or tanshinone (10 μM) for 30 min prior to adding LPS (10 μg/ml) and incubating for an additional 2 h. The assay to determine AP-1 luciferase activity was performed as described previously [20].

### Immunofluorescence staining

RPMI-2650 cells were grown on glass coverslips in 6-well plates for 24 h. Before fixing, cells were pretreated with inhibitors for 1 h, followed by treatment with LPS (10 μg/ml) for 1 h. The treated cells were fixed with 4% formaldehyde for 10 min and permeabilized with 0.1% Triton X-100 in PBS at 4°C for 10 min. After blocking with 3% BSA in PBS at 37°C for 30 min, cells were probed with anti-phospho-c-Jun (Ser63, Cell Signaling) and Alexa Fluor 488-conjugated anti-mouse antibody (Jackson ImmunoResearch Laboratories, West Grove, PA). DAPI (0.2 μg/ml) was used for nuclear staining. The stained cells were then analyzed under a fluorescence microscope (Carl Zeiss, Göttingen, Germany) with an objective (oil immersion, aperture 1.3) of magnification 63×.

### Patient selection and ethics statement

In total, 12 patients with CRSwNP, enrolled in this study had symptoms of mucopurulent discharge with nasal obstruction. The purulent discharge and nasal polyps were collected from middle or upper nasal meatus as described previously [21]. The patients underwent sinus surgery because of poor response to drug therapy, defined as at least 3 months of poor response to empirical antibiotics [22]. During endoscopic sinus surgery, we obtained pus samples from the location of discharge in the ostiomeatal complex or upper nasal meatus, using an aseptic cotton microtip. Positive results from bacterial culture of these samples confirmed sinus infection [23]. This study was specifically approved by the Institutional Review Board of the China Medical University Hospital (approval number: DMR101-IRB1-135, Taichung, Taiwan). Each participant signed the written consent form before the samples were obtained.

### Immunohistochemistry (IHC) analysis

Six CRSwNP of eosinophilic type patients with or without bacterial infection were randomly selected for immunohistochemical (IHC) analysis. The expression levels of BPIFA1 and IL-13 were detected by IHC staining, as previously described [10]. The pathologist counting was evaluated the numbers of eosinophils under microscopy at high-power (HP) magnification (Å~400) with random selection of 10 HP fields. An over 10% percent eosinophils in total inflammatory cells was defined as the eosinophilic predominant CRSwNP.

### Statistical analysis

The overall difference in multiple groups was analyzed by ANOVA. Post-test for ANOVA was analyzed by Tukey’s Honestly Significant Difference Test (Tukey’s test). *P* < 0.05 was considered statistically significant. Statistical analyses were carried out using the SPSS program (version 12.0; SPSS Inc., Chicago, IL, USA).

## Results

### LPS-induced BPIFA1 expression in nasal epithelial cells

**Fig 1:**
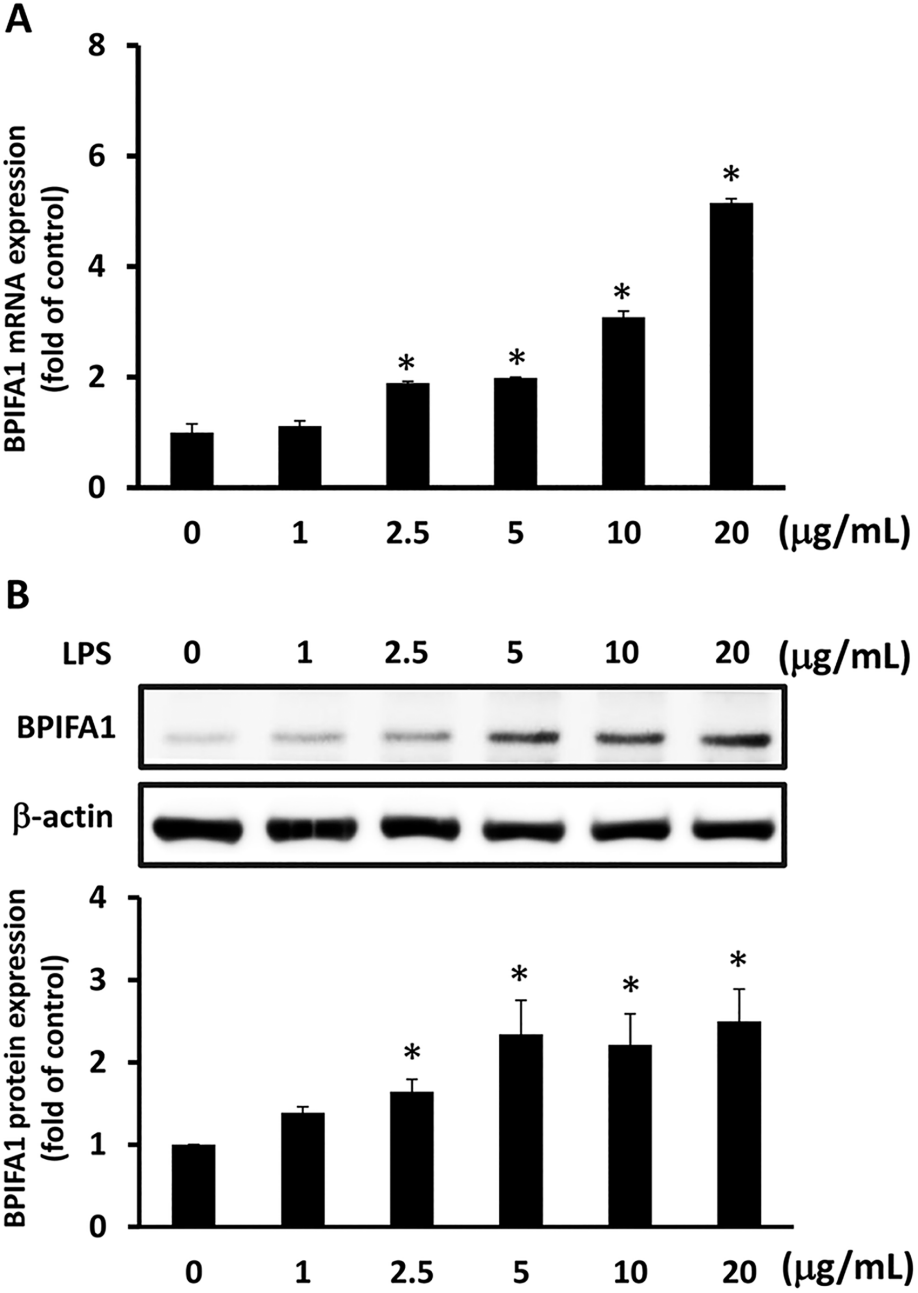
LPS induces BPIFA1 expression in nasal epithelial cells. (A) RPMI-2650 cells were treated with various concentrations (0–20 μg/ml) of LPS for 2 h, and mRNA levels of BPIFA1 were analyzed by quantitative real-time PCR. (B) Cell lysates were then prepared to measure BPIFA1 protein expression levels by western blot after incubation with LPS for 24 h. Protein expression levels of BPIFA1 were quantified by densitometric analysis and normalized to those of β-actin. ANOVA with Tukey’s test was used to compare the overall difference between the groups. *, *P* < 0.05 compared to LPS-untreated control group.

To analyze whether LPS induced BPIFA1 mRNA expression in nasal epithelial cells, RPMI-2650 cells were treated with various concentrations of LPS (0–20 μg/ml) for 2 h. Treatment of cells with LPS led to a significant increase in BPIFA1 mRNA expression level in a concentration-dependent manner (Fig 1A). We then assessed BPIFA1 protein expression in cells that were incubated with LPS at various concentrations for 24 h, by western blot. As shown in Fig 1B and S1 Fig, the expression levels of BPIFA1 protein were significantly increased by treatment of cells with L.

PS at concentrations of 2.5–20 μg/ml. These results indicate that BPIFA1 expression is induced by LPS in a concentration-dependent manner.

### Involvement of JNK/c-Jun in LPS-mediated BPIFA1 up-regulation

**Fig 2:**
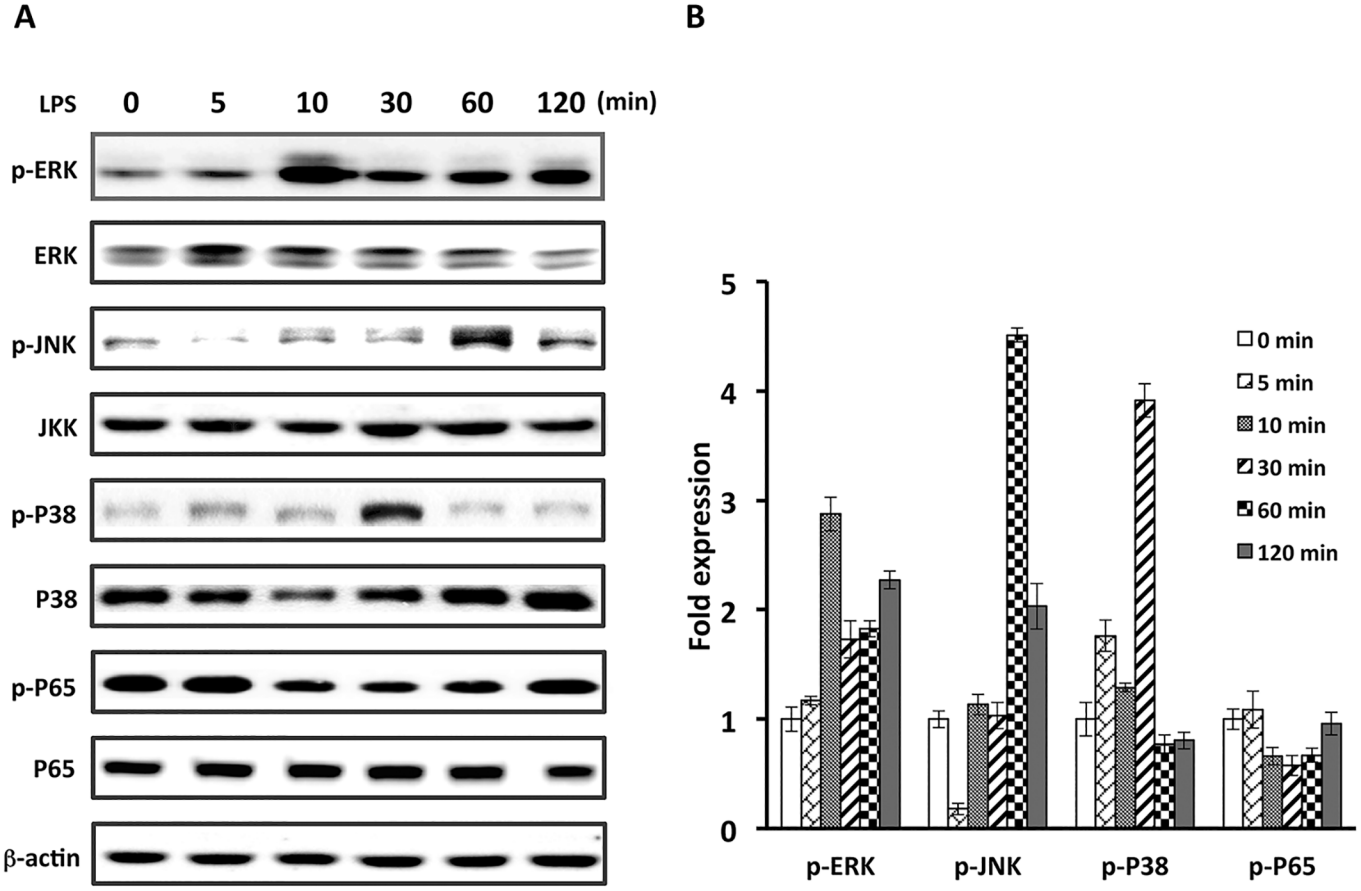
Phosphorylated p65 does not respond to LPS-mediated BPIFA1 expression. (A) RPMI-2650 cells were treated with LPS (10 μg/ml) for the indicated times, and the expression levels of phosphorylated ERK, JNK, p38, and p65 were determined by western blot. β-actin was used as the loading control. (B) Protein expression levels were quantified with densitometric analysis and presented as mean ± standard deviation, derived from three independent experiments.

**Fig 3:**
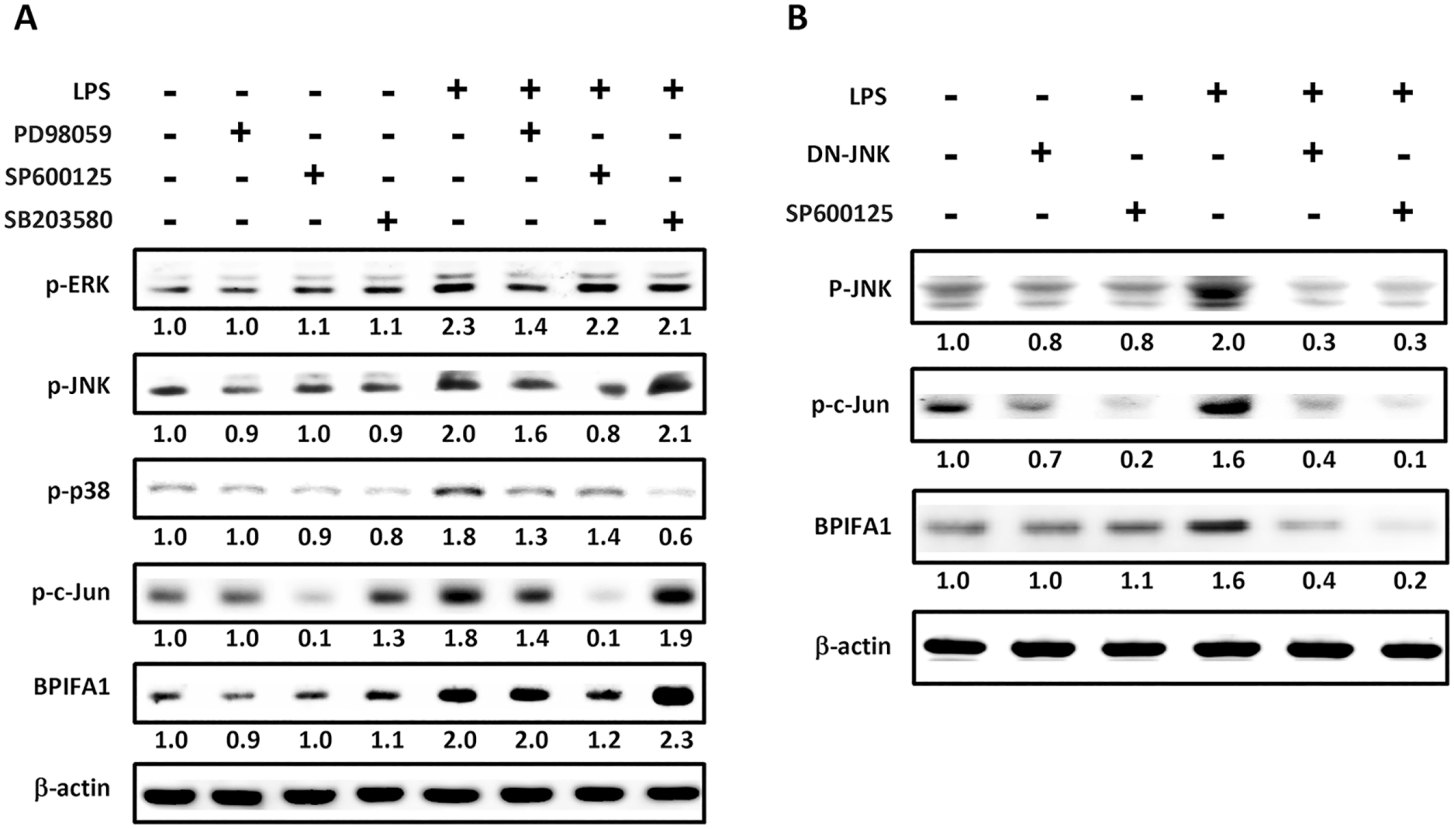
JNK/c-Jun pathway is involved in LPS-mediated up-regulation of BPIFA1 expression in nasal epithelial cells. (A) Cells were pretreated for 1 h with 20 μM PD98059 (ERK inhibitor), 10 μM SP600125 (JNK inhibitor), or 20 μM SB203580 (p38 inhibitor) and incubated with 10 μg/ml LPS for 2 h. (B) Cells were transfected with a JNK-dominant negative (DN-JNK) mutant for 24 h or pretreated with SP600125 for 30 min prior to incubation with LPS for 1 h. The protein expression levels were determined using western blot. β-actin was used as the loading control. The western blots were carried out independently in triplicate and results were representative of one of three independent experiments. The expression level of each protein was quantified by signal intensity and was indicated at the bottom of each lane. The quantitative analysis of western blot for three independent experiments was shown in S2 Fig. ANOVA with Tukey’s test was used to compare the overall difference between the groups. *P* < 0.05 was considered statistically significant.

We then determined the signaling pathway involved in LPS-induced BPIFA1 up-regulation in nasal epithelial cells. Treatment of cells with 10 μg/ml LPS stimulated the time-dependent phosphorylation of ERK, JNK, and P38 (Fig 2). The phosphorylation of ERK, P38, and JNK peaked at 10 min, 30 min, and 60 min, respectively. However, upon treatment of cells with LPS at different time points, the phosphorylation of p65 did not change. To confirm which molecules were involved in LPS-induced BPIFA1 expression, several specific inhibitors against signal molecules were employed. As shown in Fig 3A, induction of BPIFA1 by LPS was suppressed by SP600125, specific inhibitor of JNK, but not by PD98059 and SB203580, specific inhibitors of ERK and P38, respectively. Noticeably, SP600125 completely inhibited LPS-induced c-Jun phosphorylation, which is a downstream signaling molecule of JNK (Fig 3A and S2 Fig). Additionally, LPS-induced BPIFA1 expression was inhibited by SP600125. We then confirmed that JNK/c-Jun pathway plays a critical role in LPS-elicited BPIFA1 expression in nasal epithelial cells. As shown in Fig 3B and S2 Fig, treatment of cells with SP600125 reduced not only LPS-induced JNK and c-Jun phosphorylation but also BPIFA1 expression. Further, transfection with dominant-negative JNK (DN-JNK) decreased LPS-induced JNK and c-Jun phosphorylation, followed by attenuation of BPIFA1 expression (Fig 3B and S2 Fig). Taken together, these results demonstrate that LPS upregulates BPIFA1 expression in nasal epithelial cells through the JNK/c-Jun signaling pathway.

### Involvement of the transcription factor AP-1 in LPS-induced BPIFA1 expression

**Fig 4:**
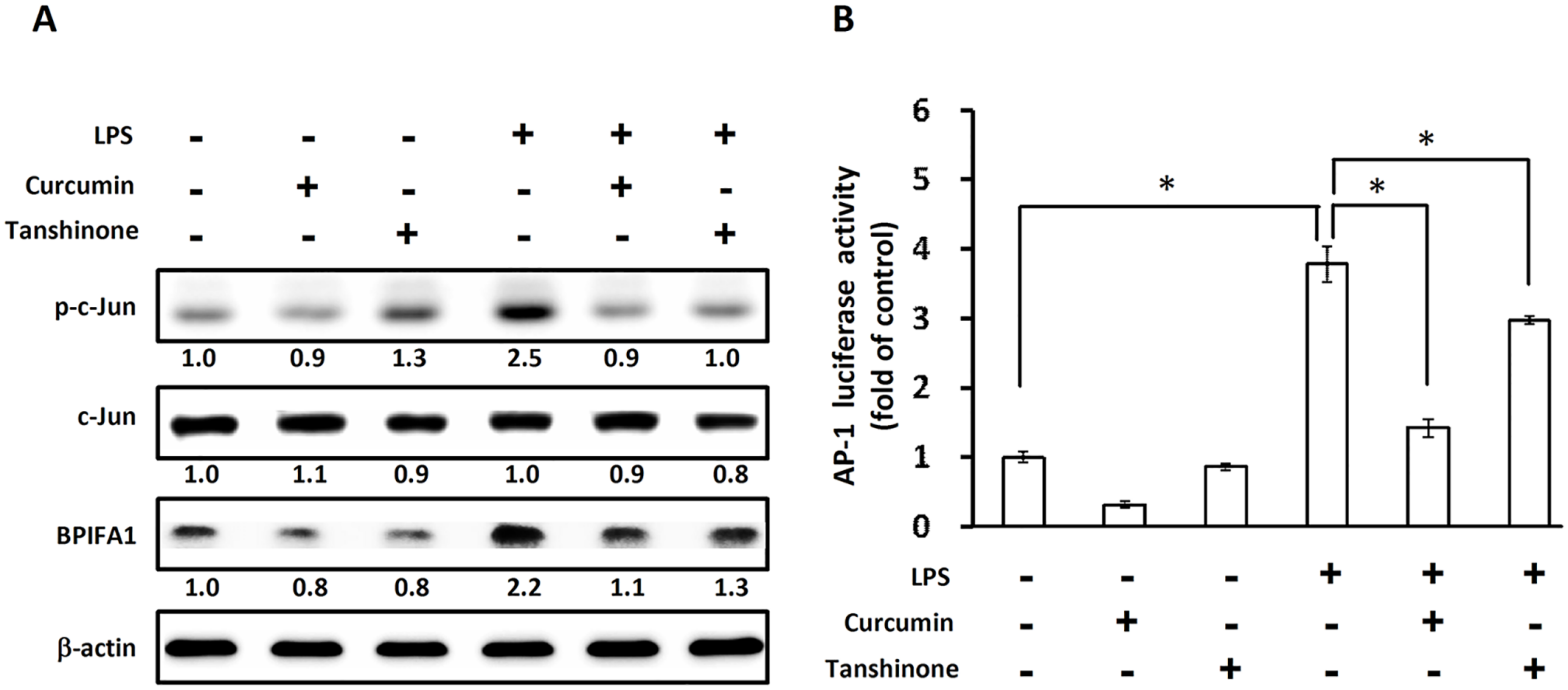
AP-1 is involved in LPS-induced BPIFA1 expression. Cells were pretreated with 10 μM curcumin or tanshinone (inhibitors of c-Jun) for 30 min, followed by incubation with LPS (10 μg/ml) for 2 h. Protein expression levels were determined using western blot and normalized to those of β-actin. The expression level of each protein is quantified by signal intensity and is indicated at the bottom of each lane. The quantitative analysis of western blot for three independent experiments was shown in S3 Fig. ANOVA with Tukey’s test was used to compare the overall difference between the groups. *P* < 0.05 was considered statistically significant. (B) Cells were transfected with AP-1-Luc reporter and incubated with LPS (10 μg/ml) for another 2 h. Cell lysates were subjected to luciferase activity assays to determine AP-1 luciferase activity. The results represented mean and standard deviation values from three independent experiments. *, *P* < 0.05 compared between two groups.

**Fig 5:**
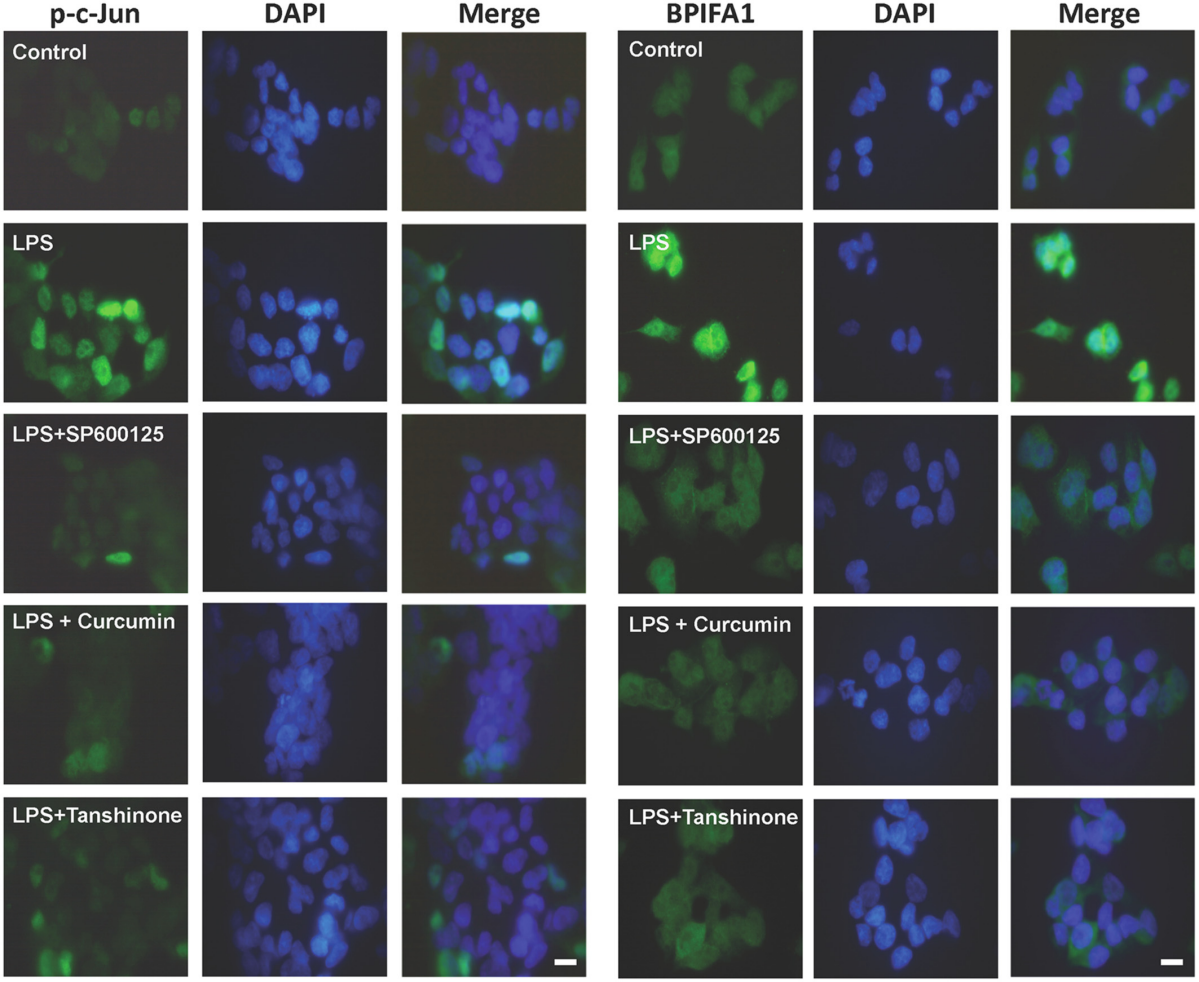
Inhibition of c-Jun suppresses LPS-induced BPIFA1 expression. Cells were pretreated with 10 μM of SP600125, curcumin, or tanshinone (inhibitors of c-Jun) for 30 min, followed by incubation with LPS (10 μg/ml) for 2 h. Cells were washed and treated with antibodies against (A) phosphorylated c-Jun or (B) BPIFA1, followed by incubation with FITC–conjugated anti-mouse IgG (green). Cells were probed with DAPI to visualize the nucleus (blue) and analyzed by confocal fluorescence microscopy. Bars, 10 μm.

Curcumin and tanshinone have been identified as inhibitors that suppress not only c-Jun phosphorylation, but also AP-1 function [24,25]. It has also been reported that inhibition of the binding of c-Jun/AP-1 to its cognate motif was responsible for the inhibition of c-Jun/AP-1-mediated gene expression [24]. To confirm that c-Jun/AP-1 play a critical role in LPS-induced BPIFA1 expression, curcumin and tanshinone were used. Treatment of cells with curcumin and tanshinone dramatically decreased LPS-induced c-Jun phosphorylation and BPIFA1 expression (Fig 4A and S3 Fig). We then investigated whether transcription factor AP-1 is involved in the enhancement of BPIFA1 expression induced by LPS. Cells were transfected with an AP-1 reporter construct (AP-1-luc) and examined for luciferase expression following treatment with inhibitors and LPS. As shown in Fig 4B, LPS treatment significantly increased AP-1 reporter activity. However, curcumin and tanshinone effectively antagonized the upregulation of AP-1 luciferase activity. We next examined c-Jun phosphorylation and BPIFA1 expression in nasal epithelial cells. As shown in Fig 5, treatment of cells with LPS enhanced the fluorescent intensity of phosphorylated c-Jun and the amount of BPIFA1 expressed. However, pretreatment of cells with inhibitors (SP600125, curcumin, and tanshinone) resulted in a marked inhibition of c-Jun phosphorylation in the nucleus (Fig 5A) and of BPIFA1 expression (Fig 5B) in the cytoplasm. These results indicate that AP-1 activation is involved in LPS-induced BPIFA1 expression in nasal epithelial cells and that this activation is mediated through the JNK/c-Jun signaling pathway.

### Attenuation of LPS-induced BPIFA1 expression by IL-13

**Fig 6:**
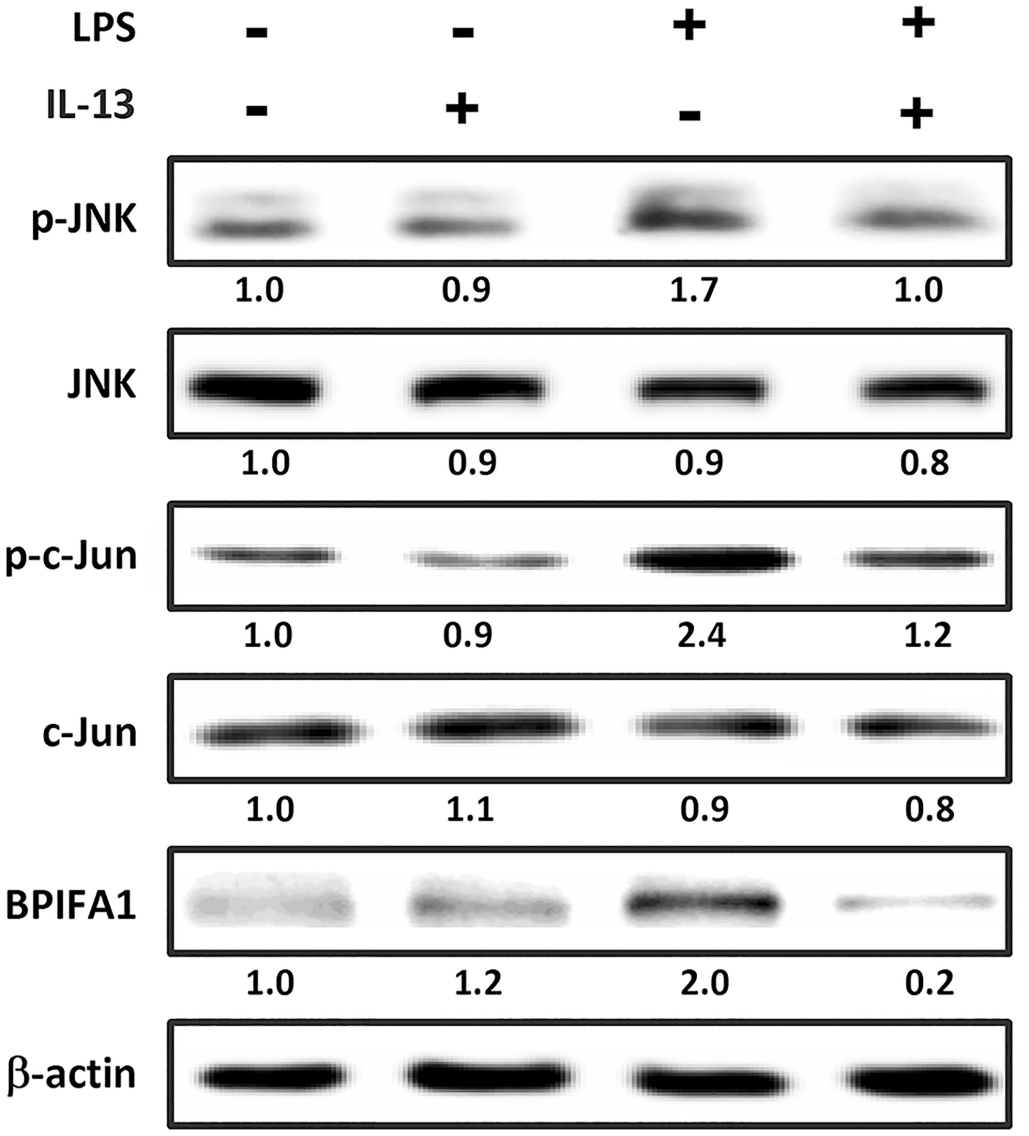
IL-13-mediates attenuation of LPS-induced BPIFA1 expression. RPMI-2650 cells, either treated or untreated with IL-13 (10 ng/ml) for 48 h, were incubated with or without LPS (10 μg/ml) for further 2 h. Cell lysates were prepared to analyze the protein expression levels by using western blot. β-actin was used as the loading control. The western blots were carried out independently in triplicate and results were representative of one of three independent experiments. The expression level of each protein was quantified by signal intensity and was indicated at the bottom of each lane. The quantitative analysis of western blot for three independent experiments was shown in S4 Fig. ANOVA with Tukey’s test was used to compare the overall difference between the groups. *P* < 0.05 was considered statistically significant.

We next investigated whether LPS-induced BPIFA1 expression could be inhibited by IL-13. Without treatment of cells with LPS, BPIFA1 was expressed at a basal level (Fig 6 and S4 Fig). Incubation with LPS alone led to an increase in phosphorylation of JNK and c-Jun, whereas treatment with IL-13 effectively suppressed LPS-stimulated phosphorylation of JNK and c-Jun, as well as BPIFA1 expression. Taken together, these results suggest that IL-13 inhibits LPS-induced BPIFA1 through the JNK/c-Jun signaling pathway.

### Decreased BPIFA1 expression was associated with IL-13 secretion in eosinophilic CRSwNP patients with bacterial colonization

**Fig 7:**
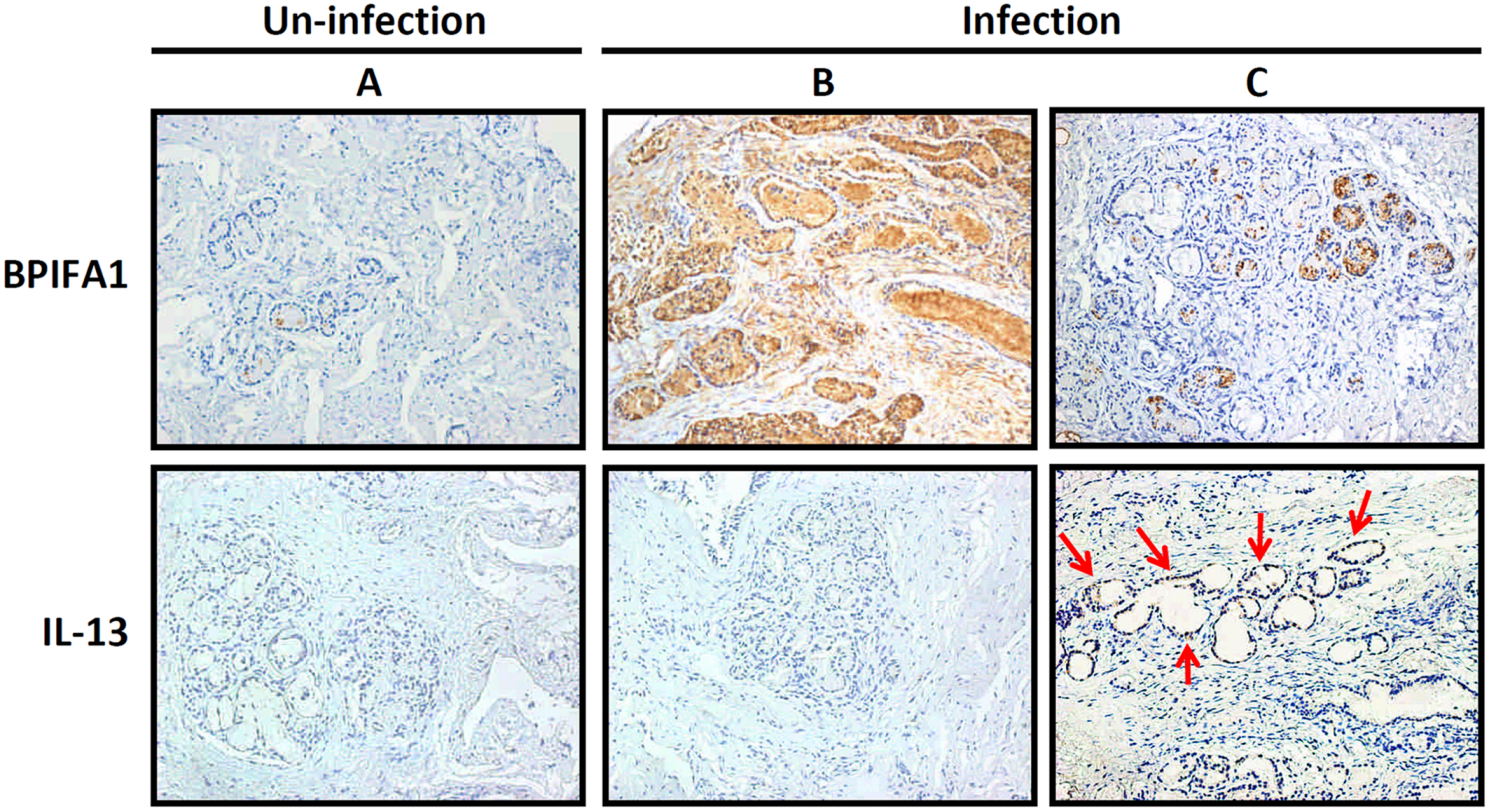
Bacterial infection is associated with elevated IL-13 secretion and reduced BPIFA1 expression in sinonasal tissues. Representative immunohistochemical staining of BPIFA1 (upper panel) and IL-13 (lower panel) expressions in eosinophilic CRSwNP patients (A) without or (B, C) with bacterial infection. Arrows indicated the expression of IL-13 in the bacterial infection tissue (C). The images were photographed at a magnification of 200×.

We performed immunohistochemical (IHC) analysis to evaluate IL-13 and BPIFA1 expression in sinonasal tissues obtained from 12 patients with CRSwNP with eosinophilic predominant type. As shown in Fig 7A, in patients (n = 6) who were un-infected with bacteria, BPIFA1 and IL-13 were not expressed in the sinonasal tissue. BPIFA1 expression was more abundant in the glands of sinonasal tissues from patients who were infected with bacteria (Fig 7B). Conversely, the level of BPIFA1 expression was markedly reduced in tissues with bacterial infection and expressing a high level of IL-13 (Fig 7C) as compared to that in tissues without IL-13 expression (Fig 7B). Accordingly, the staining intensity of IL-13 and BPIFA1 was inversely proportional. We then scored the association between BPIFA1 and IL-13 expression levels in patients with bacterial infection. As shown in Table 1, all CRSwNP patients without bacterial infection showed neither BPIFA1 nor IL-13 expression. However, BPIFA1 expression of grades 0, 1, 2, and 3 was observed in 0, 1, 2, and 3 CRSwNP patients with bacterial infection, respectively. In contrast, there were 3, 2, 1, and 0 patients showing IL-13 staining of grades 0, 1, 2, and 3, respectively, in the CRSwNP group with bacterial infection. These results collectively demonstrate that the T_H_2-skewed cytokine IL-13 prominently suppressed BPIFA1 expression in eosinophilic CRSwNP patients with bacterial infection.

**Table 1:** Distribution of the expressions of BPIFA1 and IL-13 in CRSwNP biopsies.

	% of positive cells for BPIFA1/IL-13 (score)^b^				
Biopsies^a^	<10% (0+)	10–30% (1+)	30–50% (2+)	>50% (3+)	Total
CRSwNP without bacterial infection (n, BPIFA1/IL-13)	6/6	0/0	0/0	0/0	6/6
CRSwNP with bacterial infection (n, BPIFA1/IL-13)	0/3	1/2	2/1	3/0	6/6

^a^ Tissues from 12 patients with CRSwNP were stained for BPIFA1 and IL-13 using immunohistochemical analysis.

^b^ The case with less than 10% positive staining was determined as negative (grade 0); 10–30% as grade 1+, 30–50% as grade 2+, and more than 50% as grade 3+.

## Discussion

The etiology of CRSwNP is complicated, comprising multiple mechanisms. For instance, bacterial components can work as super-antigens that induce nasal mucosa injury and cause nasal polyp formation [26]. Mucosal breaks, decreased mucociliary function, and deficiency of immune regulators such as BPIFA1 or lactoferrin have been found to play a part in the genesis of CRSwNP [27,28]. IL-13, a T_H_2-skewed cytokine, is produced in allergic rhinitis and CRSwNP of an eosinophilic-predominant type [14]. However, the nasal epithelium of CRSwNP patients could secrete IL-13 irrespective of whether they were eosinophilic predominant or non-eosinophilic predominant. In this study, we showed that IL-13 suppresses BPIFA1 secretion from nasal epithelium; hence, it may reduce the innate immune response countering the bacterial infection. Therefore, patients with CRSwNP or nasal allergies may be more susceptible to bacterial rhinosinusitis after an upper airway infection.

BPIFA1, a BPI family protein, has been found to not only have an anti-biofilm function but also play a role in preventing tumorigenesis of nasopharyngeal mucosa [7,29,30]. Patients with decreased BPIFA1 secretion are susceptible to bacterial infections of the upper airway, including those caused by *Haemophilus influenzae*, *Klebsiella pneumoniae*, and *Pseudomonas aeruginosa* [6,31,32,33]. These bacteria are predominant pathogens causing biofilm formation and resistant to antimicrobial agents. Since biofilm formation in the sinuses, adenoids, and palatine tonsils is frequently found in patients with CRSwNP and chronic adenotonsillitis, surgical intervention is generally employed to treat these conditions [23,34,35,36]. The need for repeated surgeries was also observed in low BPIFA1-expressing rhinosinusitis patients [10,33]. Moreover, the recurrence of CRSwNP is significantly higher in patients with nasal allergies [15,27]. The suppression of BPIFA1 by IL-13, secreted by diseased, and allergen-irritated nasal mucosa, could explain this connection [14,15]. These findings are consistent with our results that IL-13 suppressed BPIFA1 expression in CRSwNP patients with bacterial infections, revealing that eosinophilic CRSwNP patients are more susceptible to bacterial infection upon attenuation of BPIFA1 by IL-13.

**Fig 8:**
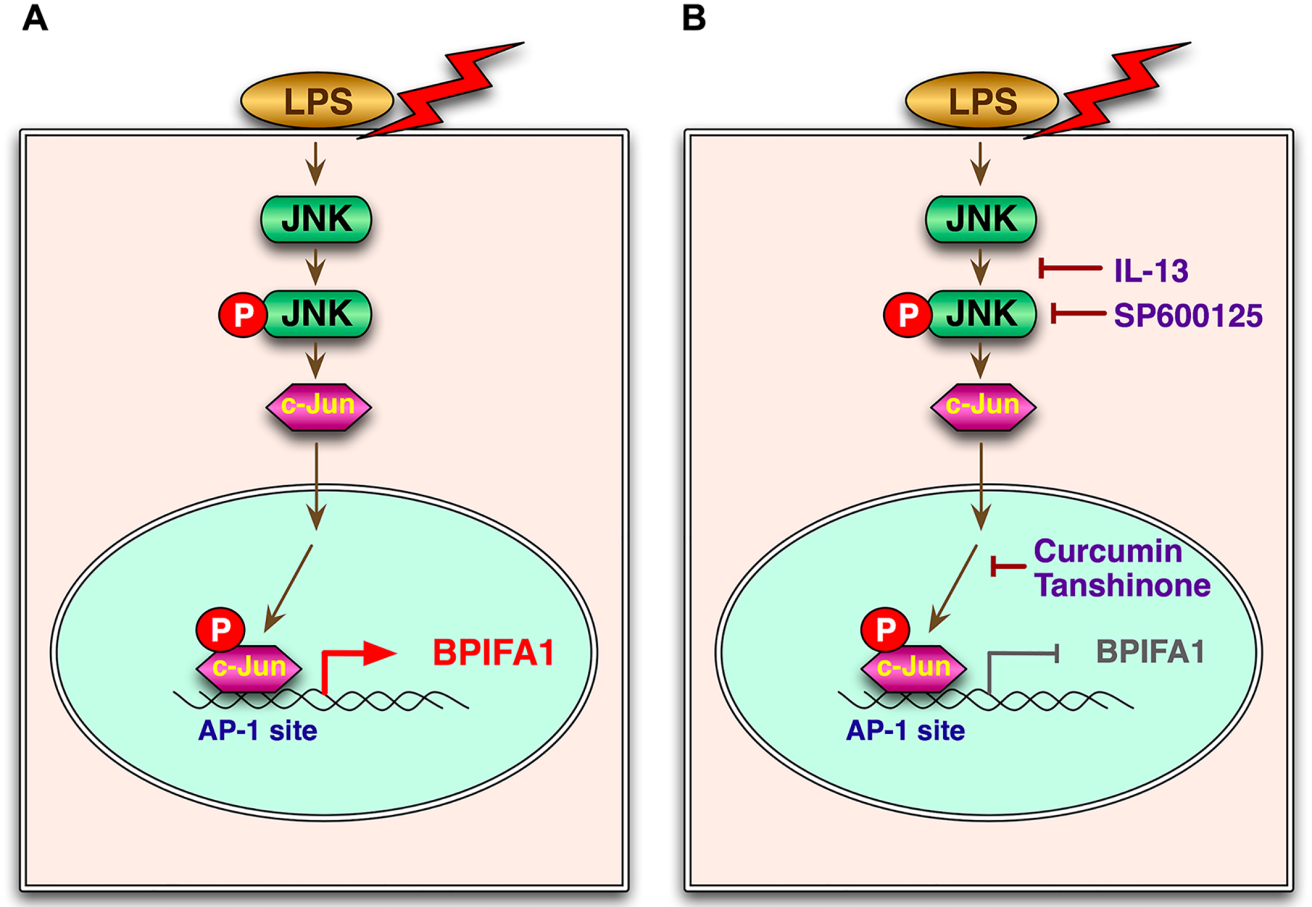
Model depicting IL-13 inhibition of LPS-induced BPIFA1 expression in nasal epithelial cells. (A) The bacterial cell wall component LPS upregulates BPIFA1 expression through the JNK/c-Jun signaling pathway, followed by AP-1 activation. (B) IL-13, a T_H_2-skewed cytokine, suppresses LPS-induced BPIFA1 expression in nasal epithelial cells from patients with eosinophilic CRSwNP.

It has been known that LPS can induce the epithelium to secrete inflammatory cytokines through the JNK and ERK pathways [37]. The suppression of JNK in the nasal epithelium may lead to the loss of wound healing functions, which is considered an etiology of nasal polyp formation in patients with rhinosinusitis [38]. These reports might support our findings that IL-13 can block LPS-induced phosphorylation of JNK and attenuate BPIFA1 expression, implicating the JNK/c-Jun signaling pathway as a major regulator in CRSwNP disease in patients with bacterial infection (Fig 8).

Our present study demonstrated that IL-13 could inhibit AP-1-related BPIFA1 production, induced by LPS-triggered inflammation in the nuclei of nasal epithelial cells. Notably, a nasal steroid spray could let AP-1 regain function to allow transcription of BPIFA1 protein at a normal level [15,27]. Therefore, nasal steroid sprays can recover BPIFA1 expression in the nasal mucosa and enhance the nasal epithelial innate immune response against LPS-related infection. This is supported by the guidelines for rhinosinusitis treatment set by European Position Paper on Rhinosinusitis (EPOS) [39] and American Academy of Otorhinolaryngology (AAO) [40], which indicated that a steroid nasal spray is the level 1 evidence for treating chronic rhinosinusitis with nasal polyps. Our results suggest a potential mechanism underlying the effect of nasal steroid sprays that not only reduces inflammation but also decreases IL-13 secretion and activates the innate function of nasal epithelium by recovering the level of BPIFA1.

Although this study has established a cell-based model to demonstrate that IL-13 suppresses BPIFA1 expression in CRSwNP patients with bacterial infections, some limitations are evident. First, the nasal samples of CRSwNP patients were positive nasal allergies with eosinophilic predominant type, we did not use nasal steroids for all of the 6 patients prior to surgery. Second, the IHC study merely demonstrated a correlation between IL-13 and BPIFA1 expression in eosinophilic CRSwNP patients. Therefore, further investigation is required to clarify the link between IL-13 production and BPIFA1 expression in other types of CRSwNP patients with bacterial infections to provide an opportunity to develop novel strategies to improve therapeutic outcomes.

## Conclusions

The results from this investigation reveal that IL-13 plays an important role in the suppression of bacteria-induced BPIFA1 expression that may be observed in patients with eosinophilic CRSwNP. Therefore, attenuating IL-13 secretion and surveying BPIFA1 production might be valuable strategies for the treatment of patients with eosinophilic CRSwNP. Future *in vivo* investigations and treatment innovations to modulate the innate immune response are warranted.

## Supporting Information

S1 Fig Uncropped pictures of Fig 1. The original uncropped western blot for protein expression of (**Figure A**) BPIFA1 and (**Figure B**) β-actin.
(PDF)

S2 Fig Quantitative analysis of western blots in Fig 3. Protein expression levels were quantified by densitometric analysis. The data were presented as means ± standard deviations for three independent experiments. ANOVA with Tukey’s test was used to compare the overall difference between the groups. *, *P* < 0.05 compared to LPS-treated alone group.
(PDF)

S3 Fig Quantitative analysis of western blots in Fig 4A. Protein expression levels were quantified by densitometric analysis. The quantitative results represent the means and standard deviations for three independent experiments. ANOVA with Tukey’s test was used to compare the overall difference between the groups. *, *P* < 0.05 compared to LPS-treated alone group.
(PDF)

S4 Fig Quantitative analysis of western blots in Fig 6. Protein expression levels were quantified by densitometric analysis. Statistical significance was determined for three independent experiments. ANOVA with Tukey’s test was used to compare the overall difference between the groups. *, *P* < 0.05 compared to LPS-treated alone group.
(PDF)
