# Supplementary figures and images for: Interleukin-13 Inhibits Lipopolysaccharide-Induced BPIFA1 Expression in Nasal Epithelial Cells
Source: PLoS One. 2015 Dec 8;10(12):e0143484. doi: 10.1371/journal.pone.0143484 (PMC4672888; doi:10.1371/journal.pone.0143484)

S1 Fig.

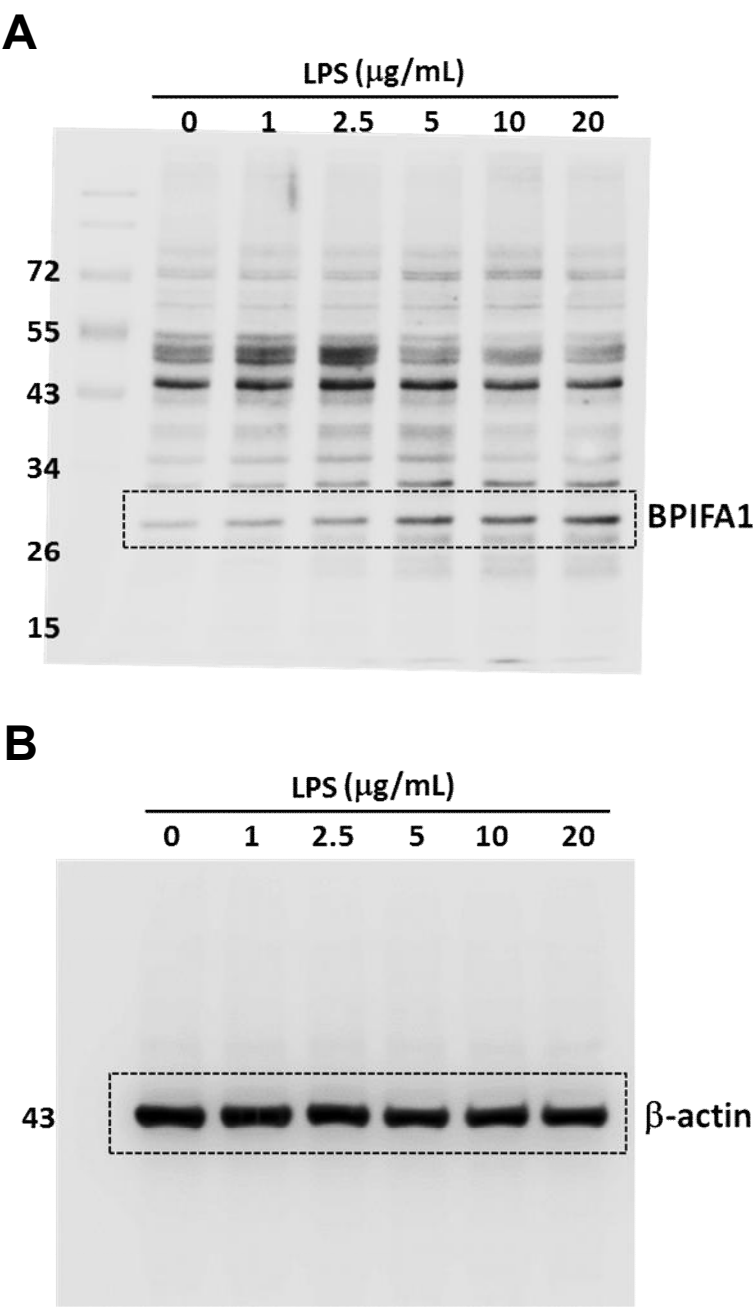

Supplement: S1 Fig — The original uncropped western blot for protein expression of (Figure A) BPIFA1 and (Figure B) β-actin. (PDF) [file pone.0143484.s001.pdf]

S2 Fig.

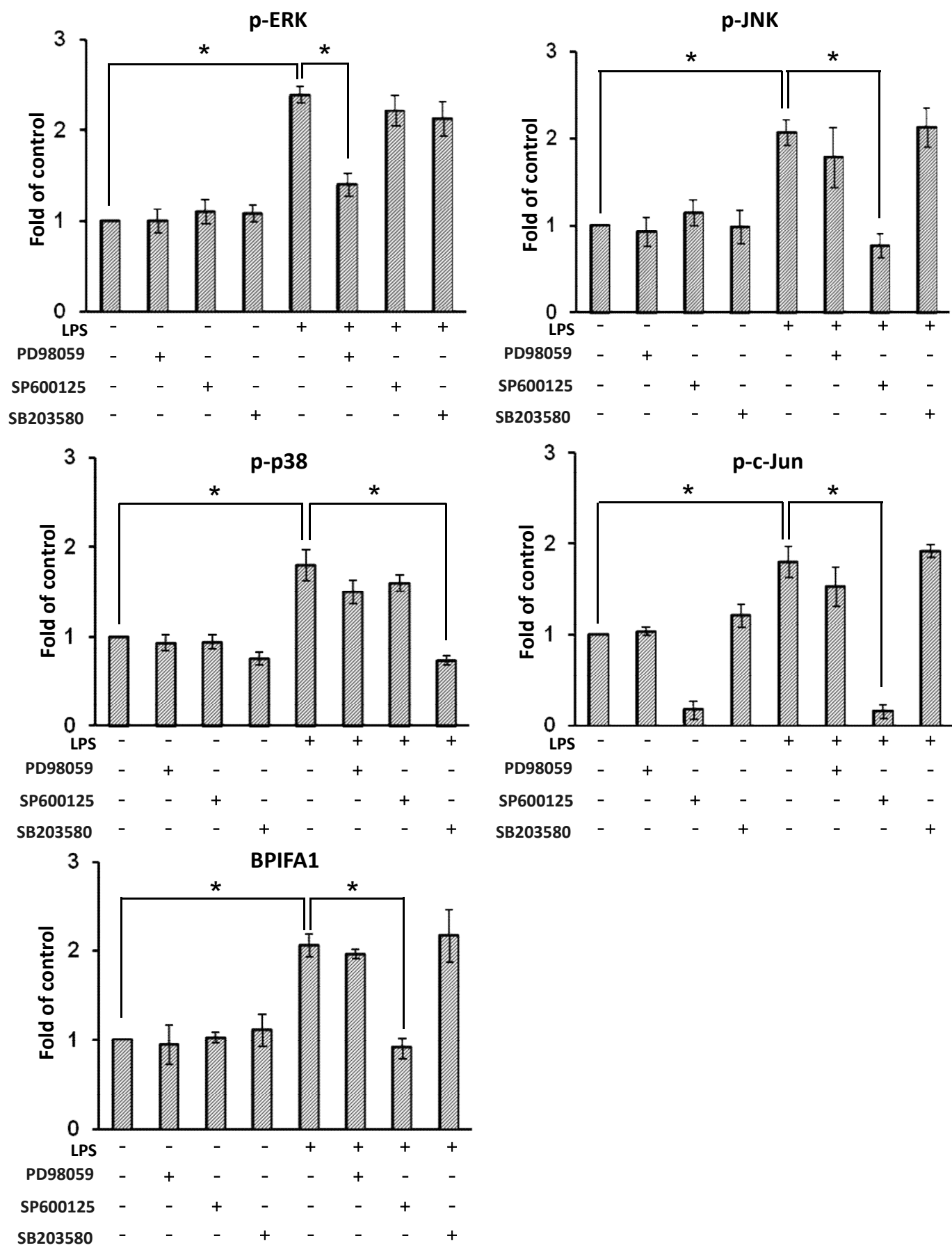

S2 Fig. continue

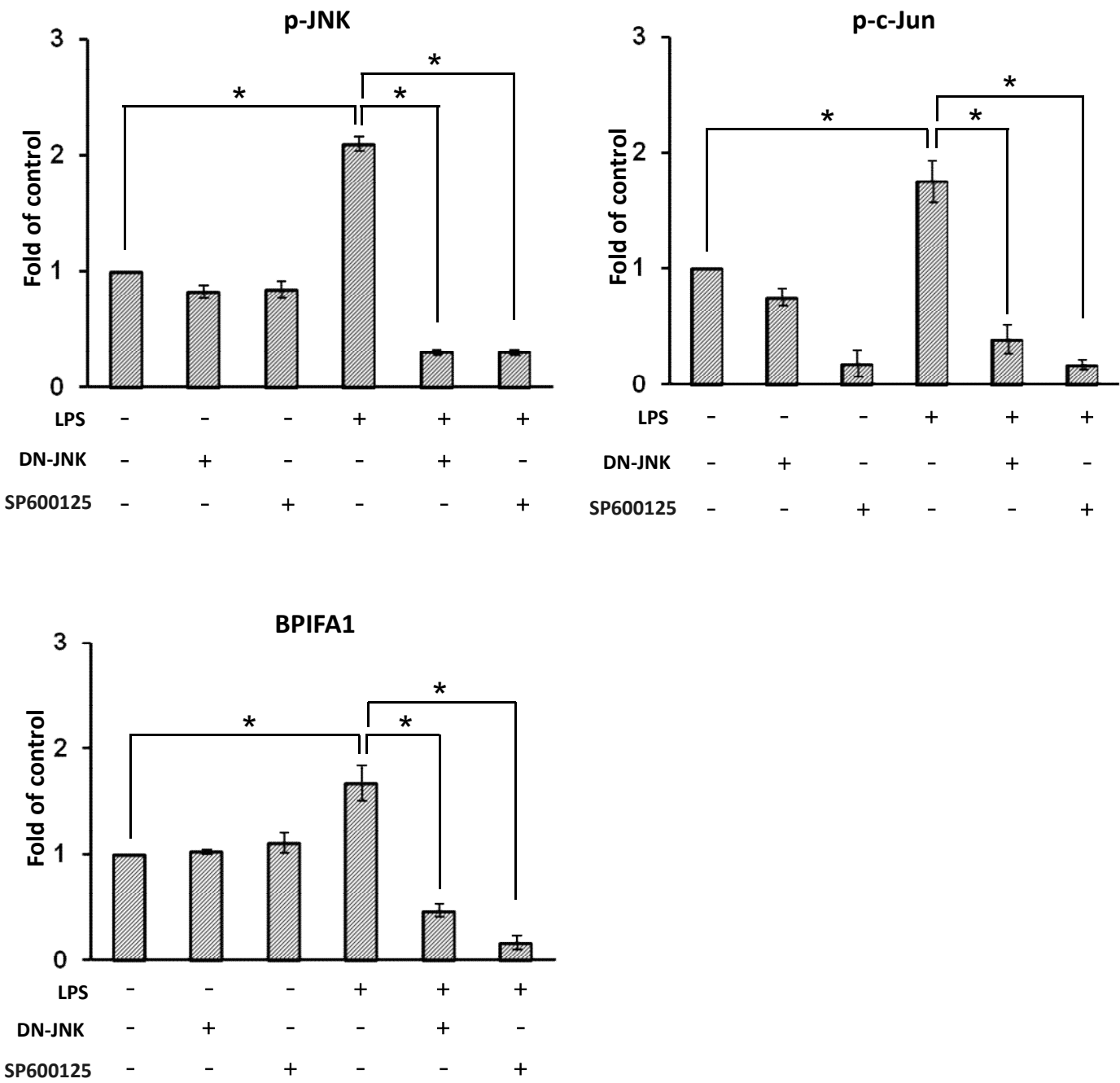

Supplement: S2 Fig — Protein expression levels were quantified by densitometric analysis. The data were presented as means ± standard deviations for three independent experiments. ANOVA with Tukey’s test was used to compare the overall difference between the groups. *, P < 0.05 compared to LPS-treated alone group. (PDF) [file pone.0143484.s002.pdf]

S3 Fig.

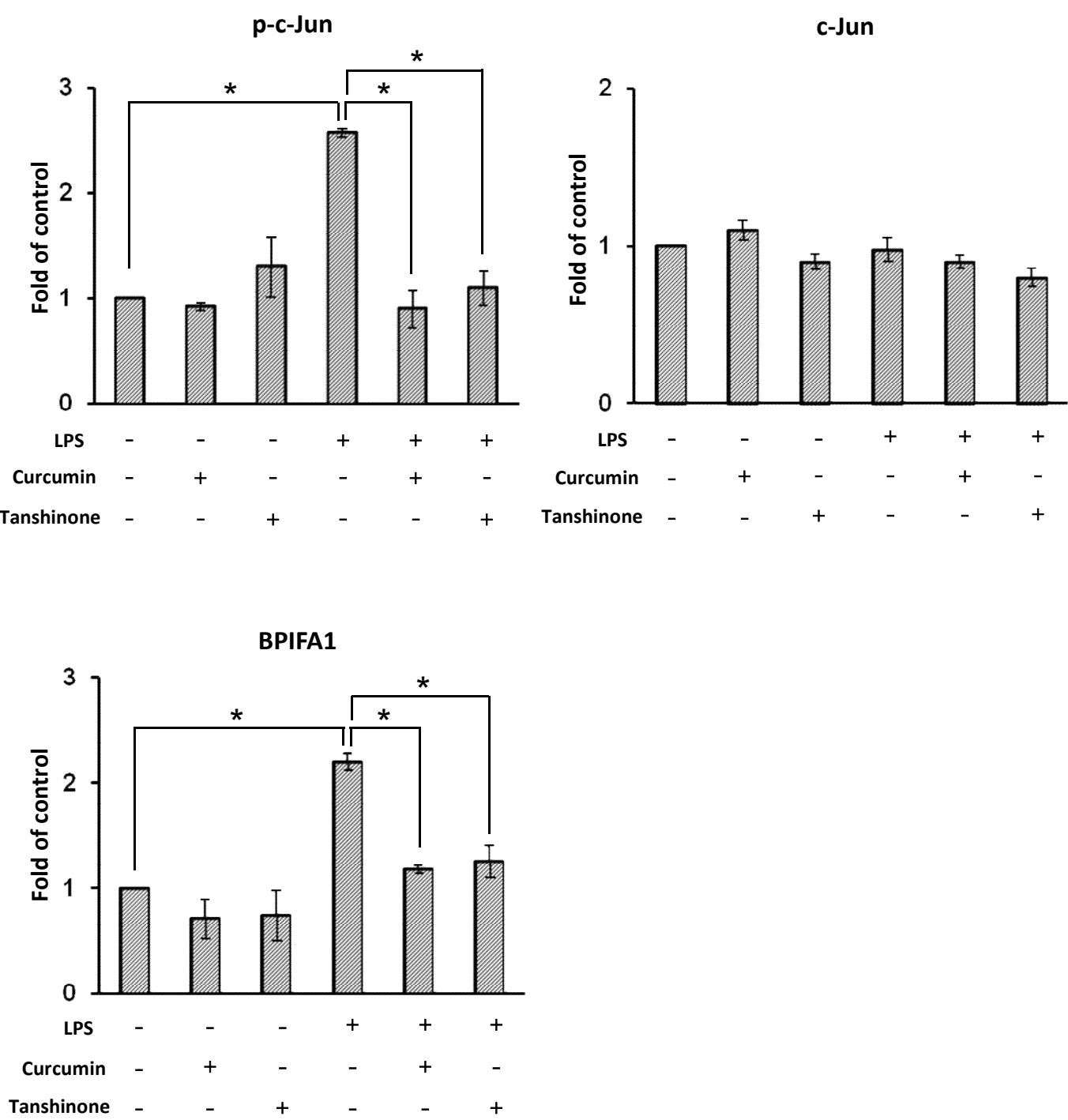

Supplement: S3 Fig — Protein expression levels were quantified by densitometric analysis. The quantitative results represent the means and standard deviations for three independent experiments. ANOVA with Tukey’s test was used to compare the overall difference between the groups. *, P < 0.05 compared to LPS-treated alone group. (PDF) [file pone.0143484.s003.pdf]

S4 Fig.

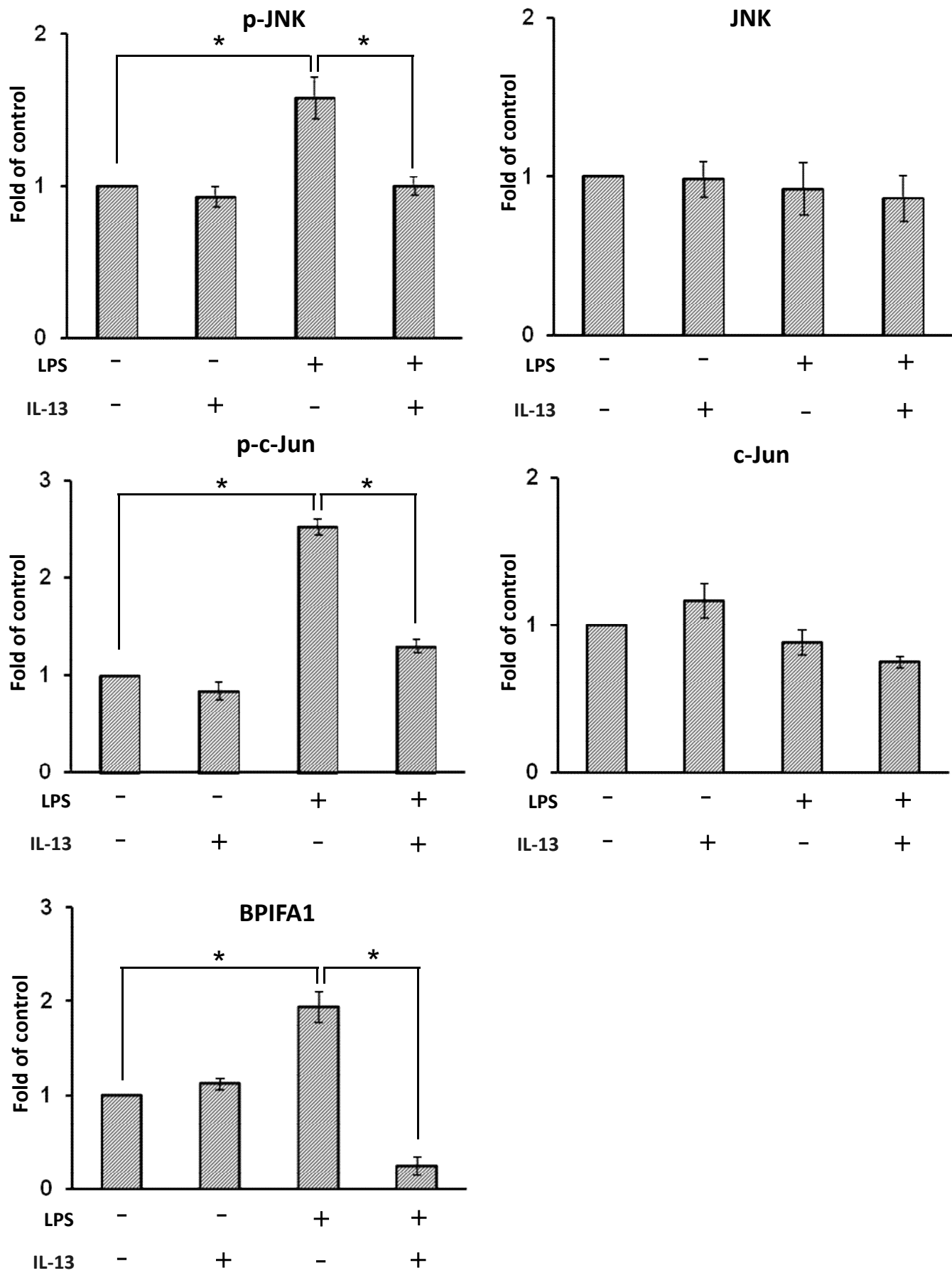

Supplement: S4 Fig — Protein expression levels were quantified by densitometric analysis. Statistical significance was determined for three independent experiments. ANOVA with Tukey’s test was used to compare the overall difference between the groups. *, P < 0.05 compared to LPS-treated alone group. (PDF) [file pone.0143484.s004.pdf]
